# Rapid response of omalizumab-resistant chronic urticaria to acalabrutinib

**DOI:** 10.1016/j.jdcr.2024.03.004

**Published:** 2024-03-15

**Authors:** Diamond R. Guy, Alicia Mizes, Christopher T. Richardson

**Affiliations:** a University of Rochester School of Medicine and Dentistry, Rochester, New York; b Department of Dermatology, University of Rochester Medical Center, Rochester, New York; c Division of Allergy, Immunology and Rheumatology, University of Rochester Medical Center, Rochester, New York

**Keywords:** acalabrutinib, anti-immunoglobulin E autoantibodies, Bruton’s tyrosine kinase inhibitor, chronic idiopathic urticaria, omalizumab

## Introduction

Chronic urticaria is characterized by persistent pruritic wheals and/or angioedema beyond 6 weeks and is subdivided into chronic idiopathic urticaria (CIU) and chronic inducible urticaria. CIU is thought to be related to the aberrant activation and degranulation of mast cells, in addition to basophil activation, autoimmunity, and genetics.^1^^,^^2^ International urticaria guidelines suggest first-line therapy for CIU with antihistamines, followed by omalizumab, and then cyclosporine.^1^ Alternative pharmacotherapies include sulfasalazine, mycophenolate, hydroxychloroquine, dapsone, and phototherapy.^2^ The therapeutic use of the Bruton’s tyrosine kinase inhibitor (BTKi) remibrutinib in CIU is under investigation via a randomized clinical trial.^3^ In this report, we discuss a case of CIU refractory to omalizumab and numerous immunomodulatory agents that showed a rapid response to the BTKi acalabrutinib.

## Case report

A 49-year-old woman presented with a 15-month history of poorly-controlled urticaria, characterized by more than 50 individual hives daily, associated with intense pruritus and occasional lip swelling. She denied associated angioedema, periorbital edema, shortness of breath, dysphagia, hyperhidrosis, arthralgias, fevers, or association with pressure, temperature, or stress. Her typical 7-day urticaria activity score (UAS7) was 42/42, correlating to high disease activity that negatively impacted her daily activities and sleep. She had a prior history of intermittent, short-lived episodes of hives well-controlled with antihistamines.

**Fig 1:**
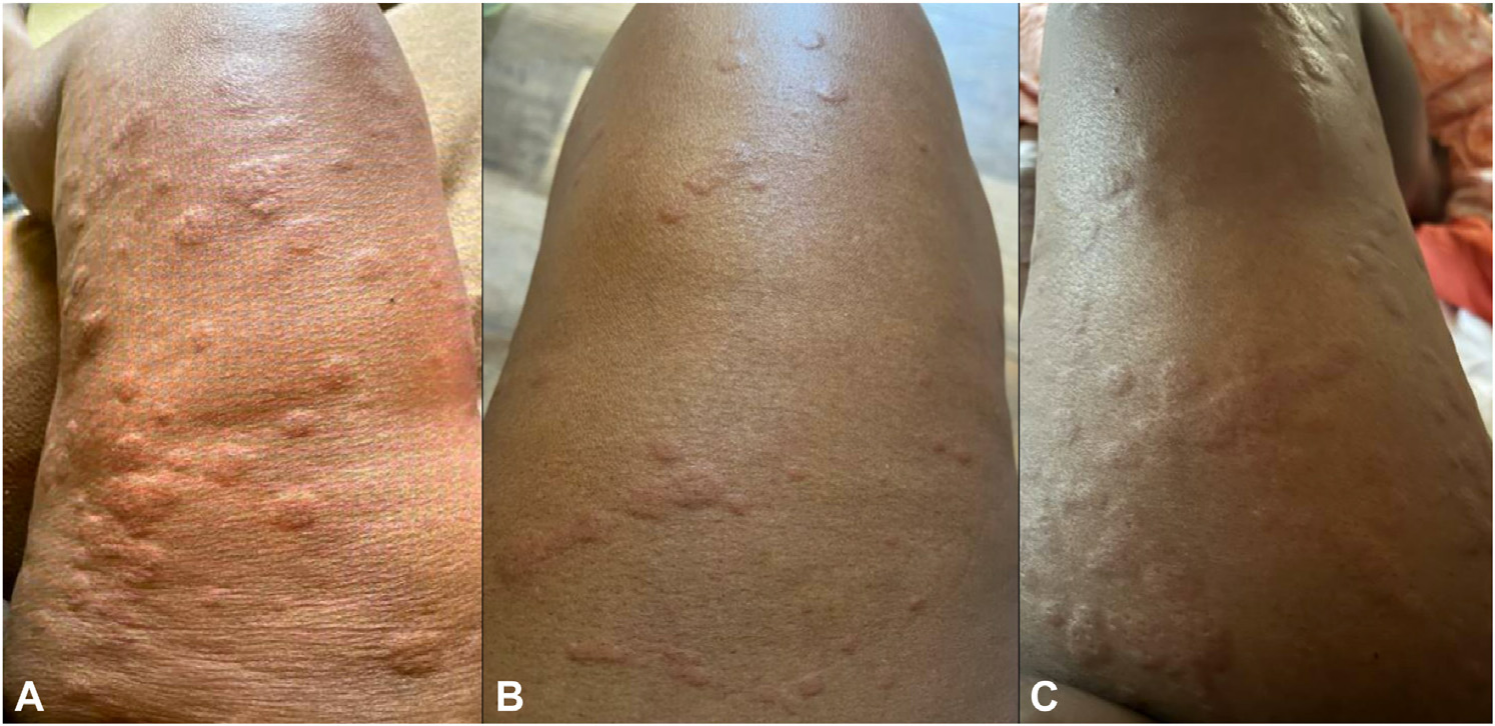
Representative images of chronic urticaria while on several failed treatment regimens. Recurrent edematous wheals on lower extremities while taking (**A**) omalizumab 300 mg every 2 weeks, dapsone 100 mg daily, and hydroxyzine 50 mg daily; (**B**) sulfasalazine 1000 mg twice daily; (**C**) fexofenadine 360 mg twice daily, omalizumab 300 mg every 2 weeks, and hydroxyzine 50 mg daily.

Physical exam showed edematous wheals with surrounding erythema involving the torso, upper extremities and lower extremities, consistent with urticaria (Fig 1). Skin biopsy revealed diffuse mild dermal edema with a perivascular and interstitial inflammatory infiltrate, predominantly of eosinophils and scattered lymphocytes, consistent with chronic urticaria. Serologic testing revealed antinuclear antibodies 1:320, elevated creatine kinase, mild transaminitis, low C1q, and low C4. Additional testing for double-strand DNA, extractable nuclear antigens, rheumatoid factor, serum protein electrophoresis, comprehensive metabolic panel, complete blood count, thyroid stimulating hormone, free-thyroxine, C1 esterase inhibitor levels and function, C3/4, hepatitis B and C, and human immunodeficiency virus was unremarkable. Autoantibodies to IgE or FcεRI (high-affinity IgE receptor) were highly positive at 46% CD203c-positive basophils (FIERA, Mayo Clinic Laboratories, reference value 0% to 12%).

The patient had failed numerous antihistamine regimens, including nonsedating antihistamines at doses of up to 4 times daily (fexofenadine 180 mg, cetirizine 10 mg, levocetirizine 5 mg) with sedating antihistamines at night (hydroxyzine 50 mg, diphenhydramine 25 mg) and cimetidine 400 mg twice daily. Omalizumab 300 mg every 4 weeks was given for about 6 months, followed by 300 mg every 2 weeks for an additional 6 months. A partial response was observed for the first 2 months only and she was unable to taper antihistamines. Her symptoms only improved with the addition of short courses of prednisone (up to 40 mg daily), which she required several times. Multiple third-line medications were trialed but discontinued due to side effects or lack of efficacy (Table I): hydroxychloroquine 200 mg daily for 4 months, cyclosporine 100 mg twice daily for 1 week, mycophenolate mofetil 1000 mg twice daily for 2 months, dapsone 100 mg daily for 1 month, and sulfasalazine 1000 mg twice daily for 3 months.

**Table I:** Prior failed treatments

Medication	Maximum dose	Duration	Efficacy/Side effects
Antihistamines			
Cimetidine	400 mg twice daily	2 y	breakthrough urticaria
Cetirizine	20 mg twice daily	15 mo	breakthrough urticaria
Diphenhydramine	25 mg daily	15 mo	breakthrough urticaria
Fexofenadine	360 mg twice daily	4 mo	breakthrough urticaria
Hydroxyzine	50 mg nightly	10 y	breakthrough urticaria
Levocetirizine	10 mg twice daily	11 mo	breakthrough urticaria
Biologic			
Omalizumab	300 mg every 2-4 wk	12 mo	breakthrough urticaria
Immunosuppressants			
Cyclosporine	100 mg twice daily	1 wk	hirsutism, hypertension (150/100 mmHg)
Dapsone	100 mg daily	1 mo	leukopenia
Hydroxychloroquine	200 mg daily	4 mo	breakthrough urticaria, hair thinning
Mycophenolate mofetil	500 mg twice daily	2.5 mo	breakthrough urticaria
Sulfasalazine	1000 mg twice daily	3 mo	breakthrough urticaria

Given her numerous treatment failures and positive FIERA test (IgG anti-FcεRI antibodies, which are inherently omalizumab-resistant), she was initiated on acalabrutinib 100 mg twice daily and had complete cessation of urticaria within 24 hours. All other medications for CIU were discontinued except hydroxyzine 50 mg nightly, which the patient continued for 3 months for sleep. She developed new symptoms shortly after starting acalabrutinib, including a headache for 2 weeks, muscle aches in the back and chest, and constipation. All were managed with over-the-counter medications. She also had a single episode of epistaxis, single heavy menstruation, COVID infection, and otitis media. After 2 months, acalabrutinib dose was reduced to 50 mg twice daily, which resulted in resolution of all side effects. She has remained hive-free for 4 months unless she skips a dose, in which case she develops a few hives that resolve with the next dose. Electrocardiogram, complete blood count, and comprehensive metabolic panel have been unremarkable.

## Discussion

CIU can considerably impact quality of life and be resistant to numerous treatments. Although omalizumab has greatly improved CIU treatment, some patients are late-responders or nonresponders, which is thought to be due to anti-FcεRI IgG and IgE autoantibodies.^4^^,^^5^ IgG autoantibodies that directly crosslink FcεRI are not blocked by omalizumab, which targets IgE. Anti-FcεRI autoantibodies promote basophil activation and subsequent upregulation of surface CD203c. These activated basophils release significantly more leukotriene C4 and are associated with IgE-independent CIU (decreased likelihood of responding to omalizumab).^6^^,^^7^ We hypothesize that our patient has IgE-independent CIU given her high percentage of active CD203c basophils and nonresponse to omalizumab. Of all of her treatments, only prednisone and acalabrutinib at all impacted her UAS7 score of 42, with acalabrutinib quickly reducing it to 0.

BTKis were initially developed for treating B-cell lymphoid malignancies, but clinical trials are exploring BTKi for other immunologic diseases including lupus, rheumatoid arthritis, atopic dermatitis, and multiple sclerosis.^8^ A randomized clinical trial demonstrated that remibrutinib is effective in patients with refractory CIU at doses of 10 mg daily to 200 mg twice daily.^3^ Remibrutinib and acalabrutinib are both oral, covalent, highly selective BTKi; acalabrutinib binds Cys481 in the ATP-binding site of BTK with an IC50 of 3 nM, whereas remibrutinib binds Cys481 with an IC50 of 1.3 nM.^8^ Furthermore, remibrutinib inhibits the degranulation of basophils and mast cells independently of FcεRI expression and omalizumab response in CIU patients.^3^^,^^6^ Similarly, acalabrutinib works via the FcεRI-BTK pathway to inhibit mast cell IgE-induced degranulation, release of histamine, leukotriene C4, and interluekin-4; and to downregulate CD63, CD164, or CD203c in basophils.^9^

Remibrutinib, acalabrutinib, and 10 other BTKis in clinical trials were developed to produce fewer of ibrutinib’s off target-effects (ie diarrhea, fatigue, musculoskeletal pain, neutropenia, anemia, headache, and bleeding).^10^ Of these, we chose acalabrutinib based on availability and insurance coverage. Our report and the remibrutinib trial indicate that small doses of BTKi can be effective for refractive CIU while minimizing side effects. We advocate that BTKis should be considered for CIU patients resistant to omalizumab, especially those with known anti-FcεRI autoantibodies with increased basophilic CD203c activation.

## Conflicts of interest

None disclosed.
